# Effects of Intradermal Therapy (Mesotherapy) on Bilateral Cervicobrachial Pain

**DOI:** 10.3390/jpm14010122

**Published:** 2024-01-22

**Authors:** Maurizio Ranieri, Riccardo Marvulli, Eleonora D’Alesio, Mariagrazia Riccardi, Maria Vittoria Raele, Laura Dell’Anna, Annatonia Fai, Giacomo Farì, Marisa Megna

**Affiliations:** 1 Department of Translational Biomedicine and Neuroscience (DiBraiN), Aldo Moro University, G. Cesare Place 11, 70125 Bari, Italy; maurizio.ranieri@uniba.it (M.R.); dalesioeleonoramaria@gmail.com (E.D.); m.riccardi12@studenti.uniba.it (M.R.); maryvi.92@hotmail.it (M.V.R.); l.dellanna3@studenti.uniba.it (L.D.); a.fai1@studenti.uniba.it (A.F.); marisa.megna@uniba.it (M.M.); 2 Department of Biological and Environmental Science and Technologies (Di.S.Te.B.A.), University of Salento, 73100 Lecce, Italy

**Keywords:** local intradermal therapy, mesotherapy, cervicobrachial pain syndrome, myofascial pain syndrome, miometric measurement, muscular stiffness, range of motion

## Abstract

Background: Mesotherapy is a procedure or a process of injecting drugs into the skin. This technique can help decrease the total drug dose due to its drug-sparing effect on the systemic route and can be utilized to treat nonspecific neck pain that occurs in the lateral and posterior neck. Methods: Ten patients with bilateral cervicobrachial pain were recruited and evaluated at T0 before treatments, T1 at the end of the treatment (42 days after T0), and T2 (72 days after T0). Assessments consisted of performing the Visual Analogue Scale (VAS) to evaluate pain evolution; a range of movement (ROM) and Bilateral trapezius’ tone, elasticity, and dynamic stiffness mensuration were performed using MyotonPro^®^. All patients underwent mesotherapy treatment in the trapezius muscles with 1 cc of Diclofenac Sodium and 1 cc of lidocaine diluted in 3 cc of saline for a total of 6 weeks. Results: VAS value statistically decreased at T1 and T2; ROM of neck flexion statistically increased at T1 and T2, and miometric tone and stiffness value statistically improved at T1 and T2. Conclusion: mesotherapy with Diclofenac Sodium reduced pain intensity and improved functional outcomes, with no significant adverse effects in patients with myofascial pain syndrome of cervicobrachial localization.

## 1. Introduction

Musculoskeletal conditions are a widespread issue that includes more than 100 different diseases, typically associated with pain and loss of function [1]. Musculoskeletal disorders are among the main causes of disability worldwide, which cause functional limitations in adult populations, with a prevalence of 4–5% in the adult population living in Canada, the USA, and Western Europe suffering from them [2]. 

Musculoskeletal pain and physical disability can reduce social functioning and absorb a large amount of health and social care resources with a well-known economic impact. In fact, it has been estimated that musculoskeletal disease accounts for 50% of absence from work and 60% of permanent work incapacity [3].

Musculoskeletal issues are common causes of neck pain, which can be treated with medication and targeted physical therapy. Nonspecific neck pain is pain that occurs in the lateral and posterior neck and is not associated with any pathognomonic signs and symptoms. Neck pain is the fourth leading cause of disability, with an annual prevalence rate that exceeds 30%, and even though acute neck pain episodes usually disappear without treatment, almost 50% of people still experience some level of pain or frequent occurrences [4,5,6,7].

Cervicobrachial pain syndrome (CBPS) refers to neck pain that has tingling, numbness, or discomfort in the arm, upper back, and upper chest, with or without an associated headache [8].

Cervical chronic pain is considered a public health problem. A high percentage of medical consultations due to muscle pain turn out to be a myofascial pain syndrome. A study conducted in 2020 by Urits et al. stated that the prevalence of myofascial pain syndrome among patients presenting to medical clinics due to pain ranges anywhere from 30 to 93% [9]. In addition to non-pharmacological approaches, drugs have traditionally been used to manage musculoskeletal pain disorders to alleviate pain, inflammation, and functional disability [3]. Systemic pharmacological therapy with analgesics and NSAIDs is often associated with adverse effects, some of which can be life-threatening. Polytherapy may involve side effects and drug–drug interactions that can be harmful, particularly for elders and those with other disorders [10,11]. Pain relief can be achieved with the most commonly prescribed medications, which are nonsteroidal anti-inflammatory drugs (NSAIDs) and acetaminophen. Diclofenac is a widely studied NSAID for pain and inflammation, including those caused by myofascial syndrome. The use of NSAIDs, commonly used to manage musculoskeletal pain, has been linked to a range of adverse effects involving the gastrointestinal, cardiovascular, and renal systems [12]. NSAIDs can also frequently cause hypersensitivity drug reactions, especially when administered systemically. The risk of adverse effects is increased due to the easy accessibility of over-the-counter drugs and the purchase of higher doses with a medical prescription. On the other hand, the use of corticosteroids can also cause other different negative effects, including hypertension, hyperglycaemia, hyperlipidaemia, weight gain, glaucoma, cataracts, gastrointestinal toxicity, osteoporosis, myopathy, avascular necrosis, immunosuppression, impaired wound healing, mood disorders, a memory deficit, and even psychosis [13]. In order to decrease systemic drug toxicity, local pharmacological therapy (e.g., interventional spine procedures, intra-articular or periarticular injections, topical administration of pharmacological agents), if effective and reliable, represents a valid tool to avoid systemic NSAIDs treatment in musculoskeletal disease management.

Local intradermal therapy (LIT), or mesotherapy, is a minimally invasive technique in which drugs are injected into the skin’s thickness, where the drug slowly spreads into the underlying tissues [14,15]. Intradermal microdeposits modify drug kinetics, retarding absorption and prolonging the local mechanism of action. It is possible to use LIT as a combined strategy to manage localized pain, especially if a systemic drug-saving effect is advantageous if it is essential to combine NSAID with other pharmacological or non-pharmacological therapies or when other therapies have failed or are not available.

A lower dosage of the drug and rapid onset with a lasting effect are the two main advantages provided by LIT treatment. The aim of the treatment is to adjust the pharmacokinetics of the injected substance and to extend the pharmacological effects in the affected region; a lower dose is enough to obtain the local pharmacological effect [10]. 

Unfortunately, the treatment protocols for pain relief are subjective, and this technique can differ based on the injected points, the drugs used (such as normal saline solution or a drug cocktail), and the dimension of the needles [16]. For example, at first, mesotherapy involved using procaine, which was delivered with multiple injections in a row (ranging from 5 to 18 injections) with needles of 30 or 40 gauge × 4 mm [17]. The current mesotherapeutic protocols require the administration of a single drug via local injection, using needles that have a 27 gauge × 4 mm or 30–32 gauge × 4 mm with the appropriate inclination to perform a microdermal deposit, with a low dose of the drug [16,18]. The gradual local spread and longer persistence of the drug in the underlying tissues make it possible to administer it at lower doses and frequencies than through the systemic route. Another important fact is given by the finding that the thickness of the dermis increases linearly with age up to 20 years and then decreases linearly afterward [19]. This results in a variation in dermal depth that can range from 1 to 1.5 mm, according to the patient [19,20,21]. Therefore, the physician should be aware of this and calibrate the depth according to the patient in front of him or her. Some future protocols should also give specific guidance depending on the age of the patient.

The advantages of LIT compared to the systemic route are as follows: increased bioavailability, drug retention around the injection site where it is needed, and limiting the adverse effects caused by the increased dose of the drug required for oral administration [20].

Patients with a minor localized musculoskeletal pain syndrome, such as neck or lower back pain, should be candidates to undergo this medical rehabilitation technique [22,23]. 

Moreover, neck pain can be treated with mesotherapy in both acute and chronic conditions. 

The aim of this study is to assess the usefulness of mesotherapy on cervical pain and stiffness.

## 2. Materials and Methods

Data collection was conducted between September and October 2023.

### 2.1. Patients Characteristics

With the aim of obtaining a homogeneous group of patients, we established inclusion and exclusion criteria so that the characteristics of patients could be known to all. Specifically, inclusion criteria were as follows: bilateral cervicobrachial pain with trapezius muscle contracture; age > 18 years; a Visual Analogue Scale (VAS) value greater than 7 for at least 15 days and still present at the moment of recruitment into the study; no targeted therapy within a month prior to enrollment such as steroids, NSAIDs, pain relievers, etc.; patients are able to comprehend the aim of the study and the possible side effects of administering the therapy, and are able to sign the informed consent. On the other hand, the following characteristics were considered to exclude patients from the study: age < 18 years; a VAS value lower than 7 for at least 15 days and at the moment of recruitment into the study; monolateral cervicobrachial pain; any other known pathology that could in any way interfere with or make doubtful the results obtained, such as in the case of rheumatological or neurological disorders affecting the cervicobrachial district; local drug infiltration (steroids) or physiotherapy such as laser therapy, physical exercise or therapy with ultrasounds, which the patient submitted to in the 45 days before recruitment; the intake of any NSAIDs or pain relievers during the month before the recruitment; the intake of any dietary supplementation or slow-release medications that could interfere with the study outcome for the last 3 months before the enrollment; women who were pregnant, breastfeeding or in a condition just before menopause without contraception; patients who lent themselves to other studies in the previous 3 months.

Patients were collected from our rehabilitation clinic; after a clinical examination, the diagnosis was confirmed with an X-ray and magnetic resonance imaging.

Ten people (middle age 50.6 ± 9.4 years) affected by bilateral cervicobrachial pain were enrolled in the study. 

Here the main demographic characteristics of the enrolled patients, with the % for each item considered (Table 1).

### 2.2. Study Methods

Here, he procedure is described as follows:

At T0, which indicates the time of recruitment, physicians carefully collected each patient’s medical history, and all patients were examined. Therefore, for each patient, the following information was collected:

- Visual Analogue Scale (VAS): it is a pain assessment scale. Its value can be a number between a minimum of 0, which represents no pain sensed, and a maximum of 10, which is the worst pain the patients ever experienced;
- Range of movement (or motion) (ROM): the extent or limit to which a part of the body can be moved around a joint or a fixed point. The ROM in the movement of the flexion is between 80° and 90° for the cervical spine. The extension is about 70°, lateral flexion ranges from 20° to 45°, and the rotation increases up to 90°, both to the left and to the right; passive ROM (pROM) was evaluated; pROM was evaluated with the patient sitting, relaxed and with the doctor behind them;
- Bilateral trapezius’ tone measured in Hertz (Hz);
- Bilateral trapezius’ dynamic stiffness measured in Newton/meters (N/m).

Muscle tone: the intrinsic tension of biological soft tissues is represented as the strength perceived by the investigator for the passive mobilization of a joint when the muscle is at rest or, more generally when the tension of the muscle is at rest. It is the measure of natural muscles’ oscillation frequency (Herz); muscle stiffness, the resistance of biological tissues to a deformation force, the sensation of pain or tightness in the muscles (Newton/meters); muscle elasticity, and the capacity of the muscle after being pulled or stretched to return to its initial length (relative unit) [24]. 

All these outcomes (tone, elasticity, and dynamic stiffness) were obtained using MyotonPRO. Specifically, the instrument is placed with a uniform pretension (0.18 Newtons) to the undercut tissues in a perpendicular position with respect to the muscle, and it gives a 15-millisecond mechanical strike at a predefined force of 0.4 Newtons, followed by a rapid discharge. This whole procedure results in the onset of muscle oscillations that are recorded by the machine. Using this equipment, the tone of the muscle and its biomechanical features are calculated as a numerical value considering the vibration reduced by the muscle. The accuracy of this measurement is well-documented in the literature [25,26]. 

In the specific case of this study, measurements were obtained with the patient lying in the prone position. The neck and the shoulders had to be fully aligned with the upper limbs completely extended. In order to improve the reliability of the measurement, landmarks were marked bilaterally on the middle third of the trapezium muscles’ surface. 

All patients underwent mesotherapy treatment in the trapezius muscles with 1 cc of Diclofenac Sodium and 1 cc of lidocaine diluted in 3 cc of saline for a total of 6 weeks. In particular, they were treated two times per week for the first two weeks and, thereafter, once a week for the remaining four weeks. Mesotherapy was performed with a 32 g (0.23 × 12 mm) needle. After disinfecting the area, with patients sitting and lying down, the same operator performed approximately 30 punctures for each muscle. 

For pain management, the use of paracetamol was permitted for a maximum of 3000 mg/die, and participants were required to record their intake in a dedicated diary. In the same diary, the patients were asked to note the intake of any other medication assumed, any dosage, and side effects. 

At the treatment’s end (T1), which followed T0 of 42 days, and one month after T1 (T2), which occurred at 72 days from the enrollment, all patients were newly subjected to the previously mentioned scale assessments with the aim of comparing the results between different times and understanding whether there was any improvement, worsening, or whether the situation remained unchanged both clinically and subjectively and functionally and objectively. 

VAS and pROM were assessed by the same examiner; by contrast, the myometric measurement was performed by two different examiners due to this measurement not being operator-dependent.

Each patient signed written consent for recruitment and handed in the diary with any therapies taken and side effects upon the completion of the study.

Ethical approval was granted by the Ethics Committee “Istituto Giovanni Paolo II” of Bari, with the approval code 903.

All the procedures were performed in accordance with the Helsinki Declaration principles. 

### 2.3. Statistical Analysis

A data analysis was performed using STATA MP17 software SAS 9.4 for PC. Continuous variables were described as the mean ± standard deviation (SD) and range with categorical variables as proportions. Student’s *t*-test for independent data was used to compare continuous variables between the groups, and the ANOVA test for repeated measures was used to compare continuous variables between the groups and detection times. Multivariate linear regression was used to assess the relationship between the difference from T2 to T1 and T0 of each individual outcome and the group (treatment vs. control); correlation coefficients were calculated with a 95% confidence interval (95% CI) indicated. A *p*-value < 0.05 was considered significant for all tests.

## 3. Results

**Figure 1:**
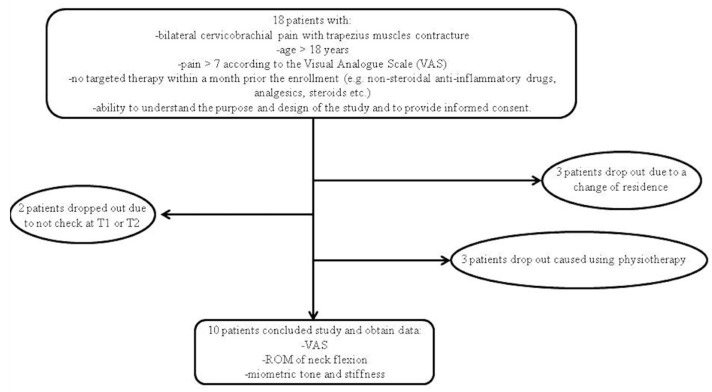
Study project. In total, 10/18 patients completed the study.

Taking into consideration the inclusion and exclusion criteria mentioned in the relevant section, a total of 18 patients were enrolled. Of these, only 10 patients completed the study by performing all the established sessions. Of the 8 that dropped out, 3 patients retired from the study due to a change in residence, 2 patients did not show up at T1 or T2, and 3 patients preferred to continue with therapeutic exercises only (Figure 1). Of the patients who completed the study, none reported any side effects.

**Figure 2:**
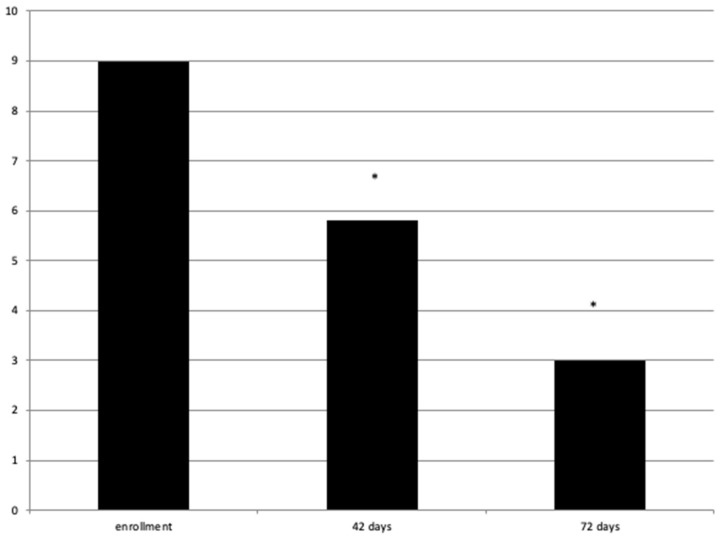
VAS value at enrollment and 42 and 72 days after. * = statistically significant.

During this study, the VAS value had a statistically significant decrease. Respectively, at T0, its value was 9 ± 0.6, at T1 = 5.8 ± 0.7, and at T2 = 3 ± 1.05 with a *p*-value < 0.05 (Figure 2).

**Figure 3:**
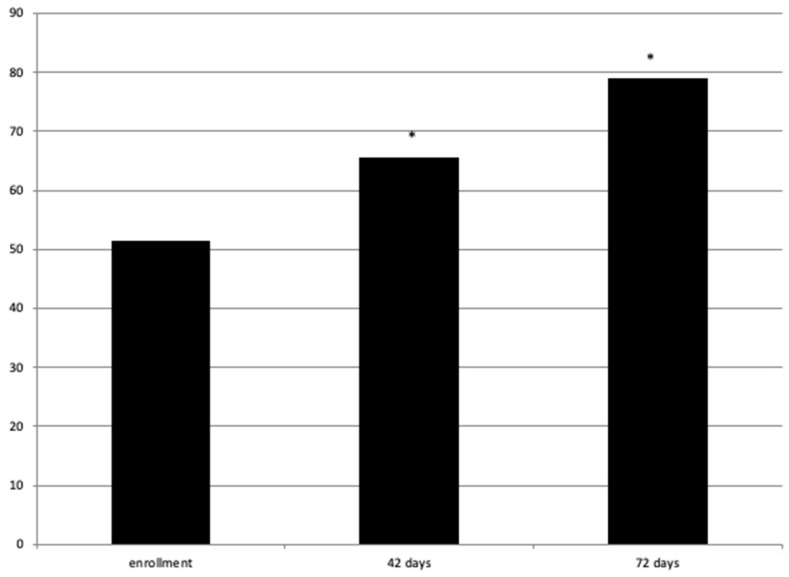
pROM of neck flexion at enrollment and 42 and 72 days. * = statistically significant.

The pROM of neck flexion at T0 (mean ± standard deviation (SD) = 51.5 ± 4.7) statistically increased at T1 (mean ± SD = 60.5 ± 5.5) and T2 (mean ± SD = 79 ± 3.9) with a *p*-value > 0.05 (Figure 3). We only measured improvements in neck flexion using a medical goniometer because it was the main problem for all patients. Other neck movements (rotation and lateral flexion) were not so disabling; however, we asked the patients if they also had any benefits from these movements, and they all gave us a positive response.

**Figure 4:**
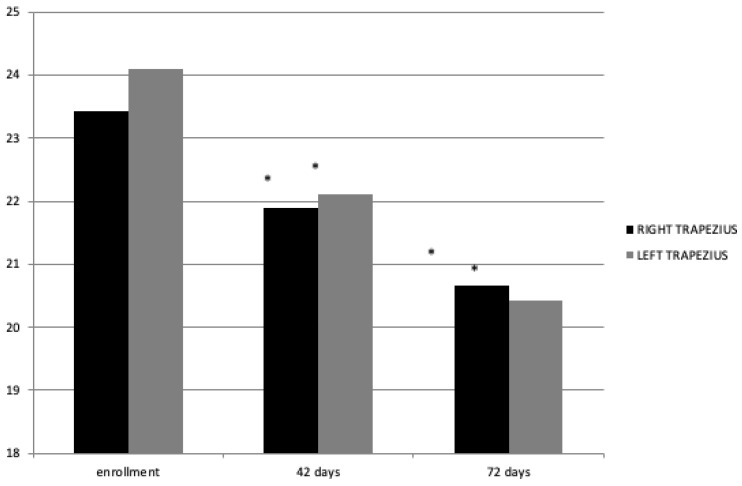
Miometric tone value (Hz) at enrollment and 42 and 72 days. * = statisticaly significant.

Figure 4 demonstrates how the miometric tone value underwent a statistically significant improvement bilaterally at T1 and T2 with a *p*-value > 0.05 (Figure 4).

**Figure 5:**
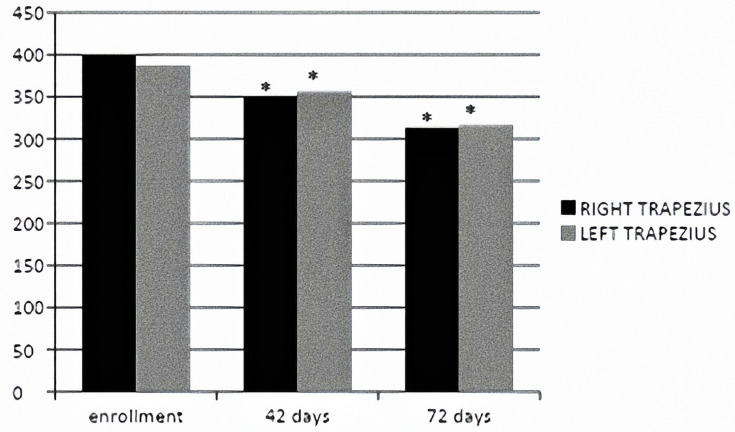
Miometric stiffness (N/m) value at enrollment and 42 and 72 days. * = statistically significant.

Figure 5 demonstrates how the miometric stiffness value underwent a statistically significant improvement bilaterally at T1 and T2 with a *p*-value > 0.05 (Figure 5).

## 4. Discussion

Chronic disorders of the locomotor system due to degenerative and functional problems have a high prevalence in Western industrialized countries. Among these, functional disorders affecting the neck region are the most common and represent a public health problem. Chronic pain in patients with musculoskeletal conditions is triggered by the inflammation and dysfunction of nerve pathways, which is known as neuropathic pain. The cervicobrachial pain syndrome is a condition in which pain in the upper quadrant is related to pain in the cervical spine. There are many different classifications that pertain to cervicobrachial pain, such as cervicobrachial pain syndrome, cervical radiculopathy, and neck and arm pain, and they are used interchangeably. As it is easy to understand from the name of the condition, the most important symptom is precisely that of pain, which is often disabling, limiting the patient’s quality of life and forcing him or her to abstain from work.

As for the pain and symptoms perceived in this region of the body and the upper limbs, these may be somatic in nature and, therefore, arise from this location, or they may be due to neuropathic mechanisms and, therefore, originate from other parts of the body but still be perceived here through the mechanism of radiation. Somatic structures include the neck muscles, zygapophyseal joint, and intervertebral discs. The nerve root or trunk is often the cause of radiating arm pain, and since one in four schwannomas originate in the nerve structures of the head and neck, it can also be a symptom of more serious pathologies for which it is necessary to make a differential diagnosis [27]. 

In the treatment of patients with chronic degenerative and functional disorders of the cervical spine, reducing pain is crucial. Also, it is known that there is a high incidence of recurrence of spinal pain. For these very reasons, aggressive non-surgical treatment, including NSAIDs, partial rest, proper posture, and body mechanics, along with home-based exercise programs, is a usual initial approach. The goal of physical therapy is to restore the function of specific muscle groups that were neglected by the patients during the course of their lives because of lack of activity or a repetitive motion/posture at work, which leads to a lack of activity in certain muscle groups and might lead to the lack of their regeneration. In some cases, cervical epidural injections or nerve root blocks may be necessary for patients to participate in physical therapy. It is believed by some evidence that trigger point injections can be more effective and more comfortable using various fluids, including water, saline, local anesthetics, vitamin B solutions, long-acting corticosteroids, acetylsalicylate, ketorolac, and the botulinum toxin, as opposed to dry needling [28,29,30]. Pain relief is often achieved with the use of NSAIDs, which are the most commonly prescribed medication. Among the NSAIDs, Diclofenac has been extensively studied for treating pain and inflammation, including myofascial syndrome. It consists of a benzene acetic derivative that stops the production of prostaglandins, which are implicated in pain and inflammation, acting as an analgesic, anti-inflammatory, and antipyretic substance [31]. It is readily available in the form of either sodium or potassium salt. In most cases, this drug is orally consumed, absorbed in the gastrointestinal tract, and eliminated by urinary and biliary excretion. After ingestion, plasma concentrations in a fasting patient tend to peak 1.5 to 2.0 h later, and its most frequent side effects affect the gastrointestinal tract, even if this is limited and in a less serious way compared with aspirin and indomethacin [32]. The use of this drug in myofascial syndrome is also well-documented in the literature. Specifically, some studies have documented the efficacy of a Diclofenac-based patch in this syndrome [33]. Moreover, a study conducted in 1986 by Frost A. showed the effectiveness of Diclofenac injections in alleviating myofascial pain compared to lidocaine injections [34].

Mesotherapy, also known as local intradermal therapy, involves injecting drugs into the thick layer of the skin [8]. The term mesotherapy derives from the French word “mesotherapie” that Michel Pistor, a French physician, coined in 1958 [35]. This study confirms that mesotherapy treatment with Diclofenac Sodium is effective for patients with cervicobrachial pain syndrome. This technique involves micro-injections of active ingredients into the skin’s surface layer, which corresponds to the area to be treated [8]. Subcutaneous drugs lead to the formation of micro-deposits, which result in the following two main benefits: a reduced dose of the active compound and a rapid start and prolonged duration of action [8]. Our observations have shown that pain control can be achieved with relatively few weekly sessions, with some patients (rapid responders) benefiting more than others. The number of sessions needed to achieve the result is probably dependent on the severity of the pain and the pathology causing it. The “mesodermal modulation” played by skin structures in response to mesotherapy could explain why certain patients respond to a single mesotherapy session while others need multiple sessions [11]. As previously mentioned, the use of this technique allows for a drug-sparing effect through the systemic route, which reduces the total drug dose [36]. In addition, an intradermal drug can diffuse into the underlying tissue and maintain tissue concentration for a longer period of time compared to when it is administered intramuscularly [8]. Since cervicobrachial pain is a condition that is as frequent as it is disabling, prompting patients to abuse drugs, we thought that mesotherapy could bring benefits to this category of patient after what was said above. In the treatment of cervicobrachial pain syndrome, mesotherapy with Diclofenac Sodium was found to be more effective at reducing pain and improving function than systemic therapy, without any additional side effects reported. These results are in accord with a previous study that showed that the systemic administration of NSAIDs is not as effective as local administration via mesotherapy [12]. Drugs that are given via mesotherapy have local effects that are close to inflammatory cells, sensory fibers, and vascular mediators. Also, micro-injections help to rebalance the nociceptive system and local actions that are not yet fully understood [25]. The pain relief reported by patients can be explained by all of these phenomena and should be supported by comprehensive studies. The mesotherapy technique’s ability to reduce pain faster has a dual benefit for patients with musculoskeletal pain in general and cervicobrachial pain specifically as follows: first, among all, is the reduction in pain itself and then the reduction in pain results in patients who are able to start physiotherapy as soon as possible with passive- and active-assisted mobilization exercises and physical therapies allowing, consequently, complete rehabilitation and the faster resolution of the problem, resulting in improved outcomes in terms of independence in daily activities. Patients showed an earlier mobilization of the involved muscles with the recovery of a cervical range of motion. The rigidity measure of the trapezius muscle determined by Myoton was also used as an outcome parameter to evaluate the effectiveness of the intervention. Data analysis revealed a significant improvement in myometric parameters following treatment. Regarding safety, there have been reports of adverse events that were caused by incorrect procedures, non-qualified personnel, or a lack of aseptic technique [37]. No patients reported any adverse effects in our study. 

In recent years, this technique has been gaining popularity in Western countries, either in combination with other therapies or alone. Its application also finds a place among treatments for cervical pain and has demonstrated promising outcomes in reducing pain and enhancing function in musculoskeletal pain disorders of the spine. In 2019, in a systematic review conducted by Paolucci et al., this procedure was studied. The following seven articles were considered: osteoarthritis of the knee (3 sessions) and pes anserine (9 sessions) were successfully treated with mesotherapy in two of them. Additionally, five studies examined spine disease, with two of them focused on chronic and nonspecific neck pain, two on acute low back pain, and one on chronic spinal pain. In one to five sessions, acute and chronic musculoskeletal vertebral pain could be fixed with the help of mesotherapy treatment [11]. In 2021, a meta-analysis showed that mesotherapy is safer and more effective than systemic therapy when it comes to treating local pain and the functional limitations caused by various musculoskeletal conditions. This study’s accuracy is limited by the heterogeneity of the injected drugs, administration technique, associated treatments, frequency, and the total number of sessions, which makes it unreproducible. However, it serves as a starting point for future studies [38]. In addition, it seems that this type of treatment is more useful and effective in acute musculoskeletal pain than in chronic pain [39]. Recent research has demonstrated that the analgesic effect can be caused not just via the injection of drugs but also by the needle and tissue trauma caused by the liquid injection into the dermis [24]. The data we have collected support a possible synergistic relationship between the pharmacological response triggered by Diclofenac Sodium injection into the dermis and the reflex analgesic mechanism triggered by the needle and saline solution. Pain can be lessened by injecting saline intradermally, but it is not as strong and lasting as infiltrating analgesic drugs [40]. These reasons have led to the hypothesis that the mesotherapy method can induce analgesic effects via pharmacological action, but also micro-traumatic effects and endocrine and neuroimmune reactions through mesodermal modulation [41].

Even in line with the available scientific literature surrounding this topic, the main limitations of our study are the small study group and the short follow-up period. Moreover, as described in the Material and Methods section (Section 2), patients could take paracetamol, indicating in a dedicated diary the dosages of the drug taken. No statistical variable monitored these aspects. Another limitation is in the study structure: it could be useful to compare the effects on cervical ROM, the VAS scale, and Myoton values obtained with injective technique or other drugs. Moreover, pain, assessed with the VAS scale, is a subjective item with can vary among populations.

## 5. Conclusions

Mesotherapy with Diclofenac Sodium reduced VAS scores and improved functional and myometric outcomes in a group of patients affected by myofascial pain syndrome of the cervicobrachial district with no significant side effects. Therefore, mesotherapy could be considered as a therapeutic alternative where classic therapies used for this condition were unsuccessful or inadvisable or in association with other treatment options. Moreover, in the short-term follow-up, it appears that the local therapy analyzed in this study might have better outcomes with respect to the systemic approach, with fewer adverse events. Its use can, therefore, be taken into consideration in standard treatment programs, but the precise algorithms need to be better investigated by scientific researchers.

Finally, we hope that further studies could better deepen our current findings involving larger samples and using increasingly effective technologies in the evaluation and cure of patients.

## Figures and Tables

**Table 1:** The main demographic characteristics of the recruited patients.

MIDDLE AGE	50.6 ± 9.4	
Patient 1	35	
Patient 2	39	
Patient 3	44	
Patient 4	45	
Patient 5	52	
Patient 6	55	
Patient 7	56	
Patient 8	57	
Patient 9	58	
Patient 10	65	
SEX		
Male	7	70%
Female	3	30%
EMPLOYMENT		
Secretary	6	60%
Employee	3	30%
Cleaner	1	10%

## Data Availability

The datasets used and/or analyzed in the current study will be made available upon reasonable request to the corresponding authors, R.M. and G.F.

## Abbreviations

LIT	local intradermal therapy
VAS	Visual Analogue Scale
NSAIDs	nonsteroidal anti-inflammatory drugs
ROM	range of motion
